# Invasive pneumococcal disease in patients from a pediatric hospital in Peru, 2017-2020

**DOI:** 10.17843/rpmesp.2022.394.12054

**Published:** 2022-12-19

**Authors:** Julio Guillermo Marín-Portocarrero, Alan Quispe-Sanchez, Flor de Maria Charca-Rodriguez, Noé Atamari-Anahui

**Affiliations:** 1 Instituto Nacional de Salud del Niño-Breña, Lima, Perú. Instituto Nacional de Salud del Niño-Breña Lima Perú; 2 Faculty of Human Medicine, Universidad Nacional Federico Villarreal, Lima, Peru. Universidad Nacional Federico Villarreal Faculty of Human Medicine Universidad Nacional Federico Villarreal Lima Peru; 3 Research Unit for Health Evidence Generation and Synthesis, Vicerrectorado de Investigación, Universidad San Ignacio de Loyola, Lima, Peru. Universidad San Ignacio de Loyola Research Unit for Health Evidence Generation and Synthesis Vicerrectorado de Investigación Universidad San Ignacio de Loyola Peru

**Keywords:** *Streptococcus pneumoniae*, Pneumococcal Vaccines, Child, Peru

## Abstract

This study aimed to describe the clinical characteristics, serotypes, and antibiotic susceptibility in patients with invasive pneumococcal disease (IPD). The medical records of patients with IPD who were hospitalized at the Instituto Nacional de Salud del Niño-Breña (Lima, Peru) were reviewed. We evaluated 29 patients. The median age was 1.9 years (interquartile range: 1 to 4 years). Of the sample, 51.7% were women and the most frequent clinical form of IPD was bacteremia in 18 (62.1%) patients; 65.5% had a complete vaccination schedule, according to the Peruvian Ministry of Health. Germ isolation was performed from blood samples in 82.8% of patients. Antibiotic resistance to erythromycin (55.2%) was the most frequent, followed by resistance to trimethoprim-sulfamethoxazole (48.3%) and penicillin (24.1%). The isolated serotypes were 6C, 19A, 23A and 24F. One patient died of meningitis. In conclusion, IPD was more frequent in children aged one to five years and the most frequent clinical form was bacteremia. Five serotypes reported in previous studies were found to be resistant to penicillin and erythromycin.

## INTRODUCTION

*Streptococcus pneumoniae* is a gram-positive, diplococcal, extracellular bacterium and it is considered the main cause of bacterial pneumonia in children, and of other diseases such as otitis media, meningitis, and sepsis ^1^. It is also one of the causes of vaccine-preventable death in children under five years of age, especially in developing countries ^2^. Although mortality has decreased by 51% from 2000 to 2015 after the introduction of conjugate vaccines ^2^, it remains a public health problem due to increasing antibiotic resistance ^3^ and the persistence of invasive pneumococcal disease (IPD) ^4^ ^,^ ^5^.

The incidence of IPD in Peru decreased from 18.4 to 5.1 cases per 100,000 children under two years of age from 2006 to 2011 ^6^, possibly due to the introduction of the heptavalent pneumococcal conjugate vaccine (PCV-7) in 2009 ^6^. Immunization with the 13-valent conjugate vaccine (PCV-13) was initiated in 2015 ^6^, so it is important to know the current status of IPD and the types of *Streptococcus pneumoniae* serotypes in the pediatric population after its implementation in the Peruvian immunization schedule.

This study aimed to describe the clinical characteristics, serotypes, and antibiotic susceptibility of patients with IPD in a pediatric hospital in Lima-Peru, from 2017 to 2020, after the introduction of PCV-13.

**KEY MESSAGES**

Motivation for the study: there are few reports describing cases of invasive pneumococcal disease after the introduction of the 13-valent conjugate vaccine in Peru.

Main findings: cases of invasive pneumococcal disease are still reported in children, more frequently in children under five years of age. The most frequent clinical form was bacteremia and there was greater antibiotic resistance to erythromycin, trimethoprim-sulfamethoxazole, and penicillin.

Implications: our findings suggest the need to maintain epidemiological surveillance of invasive pneumococcal disease and to measure the impact of vaccination against pneumococcus in children.

## THE STUDY

### Design and population

This was a descriptive and retrospective study. Data were collected from the medical records of hospitalized patients diagnosed with IPD between January 2017 and December 2020, at the Instituto Nacional de Salud del Niño-Breña (INSN-B), Lima-Peru. INSN-B is a public institution belonging to the Ministry of Health (MINSA) that provides care to pediatric patients with different diseases and also conducts research ^7^.

We included patients under 18 years of age with IPD diagnosed by *Streptococcus pneumoniae* isolation from a sterile site (blood, cerebrospinal fluid, pleural fluid, joint fluid, or peritoneal fluid) ^6^ ^,^ ^8^. We excluded cultures that were not processed at INSN-B, those that had the presence of *Streptococcus pneumoniae* plus another concomitant germ and those that were isolated from non-sterile sites (nasopharynx, pharynx, tonsils, or sputum). However, we did not find patients with these characteristics so all patients with IPD entered the study during the mentioned period.

### Study variables

We described the following variables: age at diagnosis, sex, place of origin, vaccination schedule against pneumococcus (schedule 2 + 1 was considered when the infant received the complete three doses of vaccine at 2, 4 and 12 months) and the clinical presentation: (a) pneumococcal pneumonia defined as positive blood or pleural fluid culture along with an infectious process with fever and respiratory distress as well as evidence of pulmonary infiltrates on the chest x-ray, (b) pneumococcal meningitis defined by positive cerebrospinal fluid (CSF) or blood culture along with an infectious process with fever, and signs and symptoms of neurological involvement and abnormal CSF, and c) pneumococcal bacteremia or sepsis defined by a blood culture along with systemic inflammatory response syndrome, similar to previous studies ^6^ ^,^ ^8^. Information regarding the type of culture, antibiotic susceptibility, and type of serotype was collected from the medical records of patients who had the data available. Blood agar plate was used for isolation, and identification was carried out by conventional microbiological methods based on colony morphology, alpha hemolysis, Gram staining, bile solubility and optokine susceptibility ^9^. The antibiotic susceptibility evaluation was conducted according to the 2017 guideline, Clinical and Laboratory Standards Institute (CLSI) ^10^, for different antibiotics according to availability at the institution. The Quellung reaction was used for serotyping, similar to previous studies ^6^ ^,^ ^8^. The latter procedure was carried out at the Instituto Nacional de Salud.

### Statistical analysis

Data were collected in Microsoft Excel® (Windows 2016 version). Subsequently, it was independently reviewed by two researchers (JGMP, ARQS). We analyzed the data with the STATA version 16 program (StataCorp LP, College Station, Texas, USA). Qualitative variables were described by absolute and relative frequencies, and quantitative variables by median and interquartile range (IQR).

### Ethical Aspects

The project (code PI-01/20) was approved by the Research Ethics Committee of the Instituto Nacional de Salud del Niño (N° 276-OEAIDE-INSN-2020). The present study was based on the fundamental ethical Helsinki principles; therefore, the confidentiality of the data was maintained by using a numerical code to prevent identification of the participants. Informed consent was not requested because the information was collected directly from the medical records.

## FINDINGS

We included 29 patients; the median age was 1.9 years (IQR: 1 - 4) and 51.7% were female. According to the age group, 65.5% of the patients were between one and five years old. Six patients were from other regions of Peru (Tumbes, San Martín, Ica, Junín and two from Cajamarca). Three patients had received two doses of the vaccine (age: 6, 23 and 35 months), two patients aged 5 and 25 months had received only one dose and one patient aged 8 months had received no dose. Four patients had no record of having received the vaccine. Eleven patients (37.9%) had history of antibiotic use (intravenous or oral) 30 days before hospitalization.

**Figure 1:**
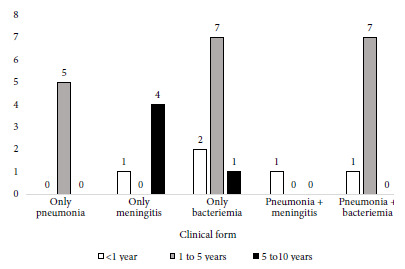
Clinical form of invasive pneumococcal disease, by age group.

One patient was under study for immunodeficiency, two had acyanotic heart disease, one had sequelae of cerebral infarction, five had iron deficiency anemia, three had renal disease (nephrotic syndrome, polycystic kidney, right hydronephrosis), four had skin disorders (three cellulitis and one epidermolysis bullosa) and three had liver disorders (hepatic glycogenosis type 3, biliary tract atresia, cytomegalovirus hepatitis). The most frequent clinical form of IPD was bacteremia in 18 (62.1%) patients, followed by pneumonia in 14 (48.3%) patients and meningitis in 6 (20.7%) patients; in some cases, two clinical forms coexisted in the same patient (Table 1). The most affected age group was the one of one to five years (Figure 1).

**Table 1:** Characteristics of patients with invasive pneumococcal disease hospitalized at the Instituto Nacional de Salud del Niño-Breña, 2017-2020.

Characteristics	n=29	%
Age (years) ^a^	1.9	(1-4)
< 1	5	17.2
1 to 5	19	65.5
5 to 10	5	17.2
Sex		
Male	14	48.3
Female	15	51.7
Place of origin (Lima)	23	79.3
Vaccination schedule 2+1	19	65.5
Clinical form		
Pneumonia only	5	17.2
Meningitis only	5	17.2
Bacteremia only	10	34.5
Pneumonia + meningitis	1	3.5
Pneumonia + bacteriemia	8	27.5
Antibiotic resistance		
Penicillin (n=29)	7	24.1
Ceftriaxone (n=28)	3	10.7
Erythromycin (n=29)	16	55.2
Vancomycin (n=29)	1	3.5
Tetracycline (n=26)	6	23.1
Trimethoprim-sulfamethoxazole (n=29)	14	48.3
Antibiotic sensitivity		
Penicillin (n=29)	17	58.6
Ceftriaxone (n=28)	17	60.7
Erythromycin (n=29)	13	44.8
Vancomycin (n=29)	27	93.1
Tetracycline (n=26)	12	46.2
Trimethoprim-sulfamethoxazole (n=29)	2	6.9
Chloramphenicol (n=9)	9	100.0

a Median and interquartile range.

Scheme 2 + 1: three doses of pneumococcal vaccine at 2, 4 and 12 months of age.

Germs were isolated from blood in 24 (82.8%) patients and from cerebrospinal fluid in five (17.2%). Antibiotic resistance was mostly reported to erythromycin, trimethoprim-sulfamethoxazole and penicillin, and sensitivity to vancomycin, ceftriaxone, and penicillin (Table 1). Only 5 (17.2%) patients had data regarding the serotype (Table 2). One two-month-old patient was registered as deceased, had meningitis, and did not have any records regarding comorbidities or serotype.

**Table 2:** Characteristics of patients serotyped for *Streptococcus pneumoniae *and hospitalized at the Instituto Nacional de Salud del Niño-Breña, 2017-2020.

Serotype	Sex	Age	Place of origin	Clinical form	Vaccination schedule	Antibiotic resistance
6C	Female	1 year, 1 month	Tumbes	Bacteriemia	2+1	Penicillin, erythromycin
19A	Female	2 years, 3 months	Lima	Pneumonia	2+1	Erythromycin, tetracycline.
19A	Female	3 years, 11 months	Lima	Bacteriemia	2+1	Erythromycin, tetracycline
23A	Female	6 months	Junín	Bacteriemia	Two doses	Erythromycin, tetracycline.
24F/ET	Male	8 months	Lima	Pneumonia/meningitis	No dose	Penicillin, erythromycin, tetracycline

Scheme 2 + 1: three doses of pneumococcal vaccine at 2, 4 and 12 months of age.

## DISCUSSION

IPD is a public health problem that continues to be a subject of research. In a period of four years, 29 IPD cases were reported after the introduction of PCV-13. The most affected the age group was the one of one to five years, despite the fact that more than 50% of the participants had completed the vaccination schedule.

The median age was 1.9 years (IQR: 1 - 4), which is higher than what was reported before the introduction of PCV-7 ^8^. Another study reported a median age of 1.17 years (IQR: 0.7 - 2.8) prior to the PCV-7, and a median age of 2.5 years (IQR: 1.2 - 7.1) after PCV-7 ^6^. In both studies, those younger than two years were the most affected ^6^ ^,^ ^8^. The fact that those around two years old are the most affected may be explained by the greater use of antibiotics and greater exposure to healthy carriers in the community ^11^.

The clinical form of bacteremia was reported in 62.1% of the patients, pneumonia in 48.3% and meningitis in 20.7%. In Peru, pneumonia and meningitis were the most frequent clinical conditions before and after the introduction of PCV-7 ^6^ ^,^ ^8^, similar to Latin American reports ^12^. The high prevalence of bacteremia may be explained by the fact that PCV-13 had already been included in the vaccination schedule during our study period, which may have reduced cases of IPD due to pneumonia and meningitis ^1^; in addition, most patients had comorbidities that may predispose to greater systemic involvement.

The isolated serotypes included in our study were 6C, 19A, 23A and 24F/ET. In Peru, before and after the introduction of PCV-7, serotypes 14, 6B, 19F and 23F were the most frequent ^6^; however, the serotypes reported by our study such as 19A, 23A and 24F, were found in smaller proportions. Serotype 6C was not reported by previous studies on IPD ^6^ ^,^ ^8^, but it has been reported in nasopharyngeal samples from healthy children ^13^ ^,^ ^14^. This serotype is relevant since it is not included in the PCV-13 and community circulation could increase the risk of IPD.

Serotypes 6C and 24F were resistant to penicillin. Penicillin resistance has been previously reported in patients with IPD ^6^ ^,^ ^8^, and more so in patients with meningitis ^15^. Likewise, all isolated serotypes were resistant to macrolides (erythromycin). In Peru, macrolide resistance in pediatric patients with IPD increased from 24.8% to 78.8% between 2006 and 2019 ^13^. The resistance mechanism is generated by the *erm* (B) and *mef* (A/E) genes, and more frequent in serotype 19A ^13^. This serotype, which we found in 2 patients, is one of the most frequent in Peru and may explain the resistance ^6^ ^,^ ^8^ ^,^ ^13^. Resistance to trimethoprim-sulfamethoxazole, penicillin, tetracycline, and macrolides has also been reported in healthy patients, so the rational use of antibiotics in outpatient care of children is important ^11^.

One patient was reported to have died from meningitis, with a case fatality rate of 3.5%. Since the introduction of PCV-7, there has been a decrease in case fatality from 22% to 7% between 2006 and 2011 in Peru ^6^, similar to what was reported in Chile ^16^. The decrease in mortality can be explained by the improvement in vaccination coverage, access to healthcare services and the introduction of pneumococcal conjugate vaccines in Peru. 

In Peru, immunization against pneumococcus began in 2009 when PCV-7 containing serotypes 4, 6B, 9V, 14, 18C, 19F and 23F was introduced into the national vaccination schedule. In 2011, the decavalent conjugate vaccine (PCV-10, PCV-7 serotypes plus 1, 5 and 7F) was introduced and by 2015, PCV-13 with PCV-10 serotypes, plus 3, 6A and 19A was introduced ^6^. We found that cases of IPD were still being reported even after the nationwide implementation of PCV-13; we report three patients with complete vaccination scheme and identified serotypes (Table 2). Serotypes 6C, 23A and 24F are not included in PCV-13; however, they are circulating in healthy carrier children ^14^. 

One of the limitations of our study is that it was carried out in a single hospital in Lima through passive surveillance, and therefore the total number of cases in Lima were underestimated. In addition, it was not possible to determine the serotype in all patients, nor did we collect data regarding the minimum inhibitory concentration. In spite of this, our results are of importance because they show the incidence of IPD after the introduction of PCV-13 in a hospital in Peru.

In conclusion, IPD was more frequent in children aged one to five years, with bacteremia as the main clinical form, and with greater resistance to erythromycin, trimethoprim-sulfamethoxazole, and penicillins. The *Streptococcus pneumoniae* serotypes found in our study have been reported by previous studies in Peru. We recommend active and passive surveillance of *Streptococcus pneumoniae* serotypes in public and private healthcare institutions in order to determine local patterns, the impact of vaccination and antibiotic susceptibility, especially after the pandemic, since vaccination coverage was affected ^17^ and the use of antibiotics was excessive, which could affect resistance mechanisms.
