# Supplementary material for: Invasive pneumococcal disease in patients from a pediatric hospital in Peru, 2017-2020
Source: Rev Peru Med Exp Salud Publica. 2022 Dec 19;39(4):469–73. doi: 10.17843/rpmesp.2022.394.12054 (PMC11397673; doi:10.17843/rpmesp.2022.394.12054)
Supplement: Translation

# Translation: Enfermedad neumocócica invasiva en pacientes de un hospital pediátrico de Perú, 2017-2020

**Authors:** Julio Guillermo Marín-Portocarrero, Alan Quispe-Sanchez, Flor de Maria Charca-Rodriguez, Noé Atamari-Anahui

**Affiliations:** 1 Instituto Nacional de Salud del Niño-Breña, Lima, Perú.; 2 Facultad de Medicina Humana, Universidad Nacional Federico Villarreal, Lima, Perú.; 3 Unidad de Investigación para la Generación y Síntesis de Evidencias en Salud, Vicerrectorado de Investigación, Universidad San Ignacio de Loyola, Lima, Perú.

**Keywords:** *Streptococcus Pneumoniae*, Vacunas Neumocócicas, Niño, Perú

## Abstract

El propósito del presente estudio fue describir las características clínicas, serotipos y susceptibilidad antibiótica en pacientes con enfermedad neumocócica invasiva (ENI). Se revisaron las historias clínicas de los pacientes con ENI hospitalizados en el Instituto Nacional de Salud del Niño-Breña (Lima, Perú). Se evaluaron a 29 pacientes. La mediana de edad fue 1,9 años (rango intercuartílico 1 a 4 años). El 51,7% eran mujeres y la forma clínica de la ENI más frecuente fue la bacteriemia en 18 (62,1%) pacientes. El 65,5% tenía el esquema de vacunación completo, según el Ministerio de Salud de Perú. El 82,8% del aislamiento del germen fue de sangre. La resistencia antibiótica fue más frecuente a la eritromicina (55,2%), trimetoprim-sulfametoxazol (48,3%) y penicilina (24,1%). Los serotipos registrados fueron 6C, 19A, 23A y 24F. Un paciente falleció por meningitis. En conclusión, la ENI fue más frecuente en niños de uno a cinco años y en la forma clínica de bacteriemia. Se encontraron cinco serotipos reportados en estudios previos con resistencia a penicilina y eritromicina.

## INTRODUCCIÓN

El *Streptococcus pneumoniae* es una bacteria grampositiva, diplococo, extracelular, considerado la principal causa de neumonía bacteriana en los niños, y de otras enfermedades como otitis media, meningitis y sepsis ^1^. También, es una de las causas de muerte prevenible por vacunación en niños menores de cinco años, especialmente en países en vías de desarrollo ^2^. Aunque la mortalidad ha disminuido entre los años 2000 al 2015 en un 51% después de la introducción de las vacunas conjugadas a nivel global ^2^, sigue siendo un problema de salud pública, por el aumento de la resistencia a los antibióticos ^3^ ^) ^y la persistencia de la enfermedad neumocócica invasiva (ENI) ^4^ ^,^ ^5^.

En Perú, la incidencia de ENI ha disminuido de 18,4 a 5,1 casos por 100 000 niños menores de dos años entre el 2006 al 2011 ^6^, posiblemente debido a la introducción de la vacuna conjugada contra el neumococo heptavalente (PCV-7) en el 2009 ^6^. El 2015 se inició la inmunización con la vacuna conjugada 13-valente (PCV-13) ^6^, por lo que es importante conocer el estado actual de la ENI y los tipos de serotipos de *Streptococcus pneumoniae* en la población pediátrica posterior a esta implementación en el calendario de inmunización del Perú. 

El objetivo del trabajo fue describir las características clínicas, serotipos y susceptibilidad antibiótica de los pacientes con ENI en un hospital pediátrico de Lima-Perú, en el periodo 2017 y 2020, posterior a la introducción de la PCV-13.

**MENSAJES CLAVE**

Motivación para realizar el estudio: existen pocos reportes que describan casos de la enfermedad neumocócica invasiva posterior a la introducción de la vacuna conjugada 13-valente en Perú.

Principales hallazgos: aún se reportan casos de enfermedad neumocócica invasiva en niños, con mayor frecuencia en menores de cinco años. La forma clínica más frecuente fue la bacteriemia y hubo mayor resistencia antibiótica a eritromicina, trimetoprim-sulfametoxazol y penicilina.

Implicancias: los hallazgos de este estudio sugieren la necesidad de mantener la vigilancia epidemiológica de la enfermedad neumocócica invasiva; además, de medir el impacto de la vacunación contra el neumococo en niños.

## EL ESTUDIO

### Diseño y población

Se realizó un estudio descriptivo y retrospectivo. Se recopilaron datos de las historias clínicas de los pacientes hospitalizados con diagnóstico de ENI entre enero del 2017 a diciembre del 2020, en el Instituto Nacional de Salud del Niño-Breña (INSN-B), Lima-Perú. El INSN-B es una institución pública que pertenece al Ministerio de Salud (MINSA) que brinda atención a pacientes pediátricos con patologías de diversas especialidades y además realiza actividades de investigación ^7^. 

Se incluyeron pacientes menores de 18 años con diagnóstico de ENI definida por el aislamiento de *Streptococcus pneumoniae* en un sitio estéril (sangre, líquido cefalorraquídeo, líquido pleural, líquido articular o líquido peritoneal) ^6^ ^,^ ^8^. Consideramos como criterios de exclusión aquellos cultivos que no fueron procesadas en el INSN-B, que tenían la presencia de *Streptococcus pneumoniae* más otro germen concomitante y que tenían aislamiento en sitios no estériles (nasofaringe, faringe, amígdalas o esputo); sin embargo, no encontramos pacientes con esas características por lo que todos los pacientes con ENI ingresaron al estudio en el periodo mencionado. 

### Variables de estudio

Se describieron variables como la edad al diagnóstico, sexo, procedencia, esquema de vacunación contra neumococo (se consideró esquema 2 + 1, cuando el lactante recibió completa las tres dosis de vacuna a los dos, cuatro y 12 meses) y la forma clínica de presentación: a) neumonía neumocócica definida como el cultivo de sangre o líquido pleural positivo en presencia de un proceso infeccioso con fiebre y dificultad respiratoria y evidencia de infiltrados pulmonares en la radiografía de tórax, b) meningitis neumocócica definida por líquido cefalorraquídeo (LCR) o hemocultivo positivo en presencia de un proceso infeccioso con fiebre, y signos y síntomas de afectación neurológica y LCR anormal, y c) bacteriemia o sepsis neumocócica definida por un hemocultivo en presencia de un síndrome de respuesta inflamatoria sistémica, similar a estudios previos ^6^ ^,^ ^8^. También se describió el tipo de cultivo, susceptibilidad antibiótica y el tipo de serotipo en aquellos pacientes que tenían registro. El aislamiento se realizó por placa de agar sangre y la identificación mediante métodos microbiológicos convencionales basados ​​en la morfología de la colonia, la hemólisis alfa, la tinción de Gram, la solubilidad en bilis y la susceptibilidad a la optoquina ^9^. La susceptibilidad antibiótica se realizó según la guía 2017, Clinical and Laboratory Standards Institute (CLSI) ^10^, para diferentes antibióticos según disponibilidad en la institución. El serotipaje se realizó mediante la reacción de Quellung similar a estudios previos ^6^ ^,^ ^8^. Este último procedimiento se realizó en el Instituto Nacional de Salud.

### Análisis estadístico

Los datos fueron recopilados en el programa Microsoft Excel® (versión para Windows 2016). Posteriormente, fue revisado por dos investigadores de manera independiente (JGMP, ARQS). Con la base de datos completa, se realizó el análisis en el programa STATA versión 16 (StataCorp LP, College Station, Texas, Estados Unidos). Se describieron las variables cualitativas mediante frecuencias absolutas y relativas y las variables cuantitativas mediante mediana y rangos intercuartílicos (RIC).

### Aspectos éticos

El proyecto (código PI-01/20) fue aprobado por el Comité de Ética en Investigación del Instituto Nacional de Salud del Niño (N° 276-OEAIDE-INSN-2020). El presente estudio se basó en los principios éticos fundamentales de Helsinki, por lo que se respetó la confidencialidad de los datos usando un código numérico para evitar la identificación de los participantes. No se solicitó consentimiento informado porque la información se recolectó directamente de las historias clínicas. 

## HALLAZGOS

En el periodo de estudio se incluyó a 29 pacientes. La mediana de edad fue de 1,9 años (RIC 1 a 4). Según grupo de edad, el 65,5% tenía entre uno a cinco años. El 51,7% fue del sexo femenino. Seis pacientes procedían de otras regiones de Perú (Tumbes, San Martín, Ica, Junín y dos de Cajamarca). Tres pacientes tenían dos dosis de la vacuna (edad: 6, 23 y 35 meses), dos pacientes de cinco y 25 meses tenían solo una dosis y un paciente de ocho meses no tenía ninguna dosis. Cuatro pacientes no tenían consignado en la historia clínica las vacunas recibidas. Once (37,9%) tenían antecedente de uso de antibiótico (endovenoso o vía oral) 30 días antes de su hospitalización.

Un paciente estaba en estudio de inmunodeficiencia, dos tenían cardiopatías acianóticas, uno tenía secuela de infarto cerebral, cinco tenían anemia ferropénica, tres presentaban enfermedades renales (síndrome nefrótico, riñón poliquístico, hidronefrosis derecha), cuatro tenian trastornos cutáneos (tres celulitis y una epidermólisis bullosa) y tres presentaron trastornos hepáticos (glucogenosis hepática tipo 3, atresia de vías biliares, hepatitis por citomegalovirus). La forma clínica de la ENI más frecuente fue la bacteriemia en 18 (62,1%) pacientes, seguida de neumonía en 14 (48,3%) pacientes y meningitis en 6 (20,7%) pacientes, pudiendo coexistir dos formas clínicas en un mismo paciente (Tabla 1). El grupo de edad más afectado fue de uno a cinco años (Figura 1).

**Tabla 1:** Características de los pacientes con enfermedad neumocócica invasiva hospitalizados en el Instituto Nacional de Salud del Niño-Breña, 2017-2020.

Características	n=29	%
Edad (años) ^a^	1,9	(1-4)
< 1	5	17,2
1 a 5	19	65,5
5 a 10	5	17,2
Sexo		
Masculino	14	48,3
Femenino	15	51,7
Procedencia (Lima)	23	79,3
Esquema vacunación 2+1	19	65,5
Forma clínica		
Solo neumonía	5	17,2
Solo meningitis	5	17,2
Solo bacteriemia	10	34,5
Neumonía + meningitis	1	3,5
Neumonía + bacteriemia	8	27,5
Resistencia antibiótica		
Penicilina (n=29)	7	24,1
Ceftriaxona (n=28)	3	10,7
Eritromicina (n=29)	16	55,2
Vancomicina (n=29)	1	3,5
Tetraciclina (n=26)	6	23,1
Trimetoprim-sulfametoxazol (n=29)	14	48,3
Sensibilidad antibiótica		
Penicilina (n=29)	17	58,6
Ceftriaxona (n=28)	17	60,7
Eritromicina (n=29)	13	44,8
Vancomicina (n=29)	27	93,1
Tetraciclina (n=26)	12	46,2
Trimetoprim-sulfametoxazol (n=29)	2	6,9
Cloranfenicol (n=9)	9	100,0

a Mediana y rango intercuartílico.

Esquema 2 + 1: tres dosis de vacuna para neumococo a los 2, 4 y 12 meses de edad.

**Figura 1:** Forma clínica de presentación de la enfermedad neu-mocócica invasiva, según grupo de edad.

El aislamiento del germen se realizó a través del cultivo en sangre en 24 (82,8%) pacientes y en cinco (17,2%) por cultivo de líquido cefalorraquídeo. La resistencia antibiótica se registró con mayor frecuencia a la eritromicina, trimetoprim-sulfametoxazol y penicilina, y la sensibilidad a la vancomicina, ceftriaxona y penicilina (Tabla 1). El serotipo se registró en cinco (17,2%) pacientes (Tabla 2). Un paciente de dos meses se registró fallecido, tenía meningitis y no registraba comorbilidades ni serotipo. 

**Tabla 2:** Características de los pacientes con serotipo para *Streptococcus pneumoniae* hospitalizados en el Instituto Nacional de Salud del Niño-Breña, 2017-2020.

Serotipo	Sexo	Edad	Procedencia	Forma clínica	Esquema de vacunación	Resistencia antibiótica
6C	Femenino	1año 1 mes	Tumbes	Bacteriemia	2+1	Penicilina, eritromicina
19A	Femenino	2 años 3 meses	Lima	Neumonía	2+1	Eritromicina, tetraciclina.
19A	Femenino	3 años 11 meses	Lima	Bacteriemia	2+1	Eritromicina, tetraciclina
23A	Femenino	6 meses	Junín	Bacteriemia	Dos dosis	Eritromicina, tetraciclina.
24F/ET	Masculino	8 meses	Lima	Neumonía/meningitis	Ninguna vacuna	Penicilina, eritromicina, tetraciclina

Esquema 2 + 1: tres dosis de vacuna para neumococo a los dos, cuatro y 12 meses de edad.

## DISCUSIÓN

La ENI es un problema de salud pública que aún sigue siendo motivo de investigación. En un periodo de cuatro años, se reportan 29 casos de ENI después de la introducción de la PCV-13, siendo los más afectados el grupo de edad de uno a cinco años, a pesar de tener el esquema de vacunación completo en más del 50% de los participantes. 

La mediana de edad fue 1,9 años (RIC 1 a 4), superior a lo reportado antes de la introducción de la PCV-7 ^8^, mediana de edad 1,17 años (RIC 0,7-2,8), y después de PCV-7, mediana de edad 2,5 años (RIC 1,2-7,1) ^6^. En ambos estudios, el grupo menor de dos años fue el más afectado ^6^ ^,^ ^8^. La edad alrededor de dos años puede explicarse por el mayor uso de antibióticos y mayor exposición a portadores sanos en la comunidad ^11^. 

La forma clínica de bacteriemia fue reportada en 62,1%, la neumonía en 48,3% y la meningitis en 20,7%. En Perú, antes y después de la introducción de la PCV-7, la neumonía y meningitis eran las condiciones clínicas más frecuentes ^6^ ^,^ ^8^, similar a reportes latinoamericanos ^12^. La bacteriemia puede explicarse por qué en nuestro periodo de estudio ya se había incluido la PCV-13 en el esquema de vacunación, pudiendo disminuir los casos de ENI por neumonía y meningitis ^1^; además, la mayoría de los pacientes tenían comorbilidades que pueden predisponer una mayor afectación sistémica. 

Los serotipos aislados en nuestro estudio fueron el 6C, 19A, 23A y 24F/ET. En Perú, antes y después de la introducción de la PCV-7, los serotipos 14, 6B, 19F y 23F eran los más frecuentes ^6^; sin embargo, en menor proporción se hallaron los serotipos encontrados en nuestro estudio como el 19A, 23A y 24F. En los estudios de ENI ^6^ ^,^ ^8^, no se logró aislar al serotipo 6C registrado en una paciente, pero se ha reportado en muestras nasofaríngeas de niños sanos ^13^ ^,^ ^14^. Este serotipo tendría relevancia, pues no se encuentra dentro de la PCV-13 y la circulación comunitaria podría incrementar el riesgo de ENI. 

Los serotipos 6C y 24F fueron resistente a la penicilina. La resistencia a penicilina se ha reportado previamente en pacientes con ENI ^6^ ^,^ ^8^, y más en pacientes con meningitis ^15^. Asimismo, todos los serotipos aislados fueron resistentes a macrólidos (eritromicina). En Perú, entre 2006 a 2019, se ha registrado un aumento de la resistencia a los macrólidos en pacientes pediátricos con ENI, de 24,8% a 78,8% ^13^. El mecanismo de resistencia es generado por los genes erm (B) y mef (A/E), y más frecuente en el serotipo 19A ^13^. En nuestro estudio, este serotipo se presentó en dos pacientes y la resistencia se puede explicar porque ese serotipo es uno de los más frecuentes en Perú ^6^ ^,^ ^8^ ^,^ ^13^. También se ha reportado resistencia al trimetoprim-sulfametoxazol, penicilina, tetraciclina y macrólidos en pacientes portadores sanos, por lo que es importante el uso racional de antibióticos en la atención ambulatoria de los niños ^11^. 

Un paciente se reportó como fallecido por meningitis, siendo la tasa de letalidad de 3,5%. Desde la introducción de la PCV-7 hubo una disminución de la letalidad del 22% al 7% entre 2006-2011 en Perú ^6^, similar a lo reportado en Chile ^16^. La disminución de la mortalidad se puede explicar por el mejoramiento en la cobertura de vacunación, acceso a servicios de salud y por la introducción de las vacunas conjugadas para neumococo en nuestro país. 

En Perú, la inmunización contra el neumococo se inició el año 2009 cuando se introdujo dentro del esquema de vacunación nacional la PCV-7 que contiene a los serotipos 4, 6B, 9V,14, 18C,19F y 23F. En el 2011, se introdujo la vacuna conjugada decavalente (PCV-10, serotipos de la PCV-7 más 1, 5 y 7F) y para el año 2015, la PCV-13 con serotipos de la PCV-10, más 3, 6A y 19A ^6^. Encontramos que después de la implementación de la PCV-13 a nivel nacional, aún se reportaron casos de ENI, siendo tres pacientes con esquema de vacunación completo y serotipos identificados (Tabla 2). Los serotipos 6C, 23A y 24F encontrados, no están dentro de la PCV-13; sin embargo, se encuentran circulando en niños portadores sanos ^14^. 

Nuestro estudio tuvo limitaciones, pues fue realizado en un solo hospital de Lima a través de vigilancia pasiva, por lo que subestimaría la totalidad de casos en Lima. Además, no se pudo tener en todos los pacientes el serotipo, tampoco registramos la concentración mínima inhibitoria. A pesar de ello, es importante porque muestra la casuística de ENI después de la introducción de la PCV-13 en un hospital de Perú. 

En conclusión, la ENI fue más frecuente en niños de uno a cinco años con forma clínica de bacteriemia con mayor resistencia a eritromicina, trimetoprim-sulfametoxazol y penicilinas. Los serotipos de *Streptococcus pneumoniae* encontrados en nuestro estudio fueron reportados en estudios previos en Perú. Recomendamos la vigilancia activa y pasiva de los serotipos de *Streptococcus pneumoniae* en las instituciones de salud públicas y privadas y de esta manera conocer los patrones locales y el impacto de la vacunación y susceptibilidad antibiótica, especialmente después de la pandemia, pues las coberturas de vacunación se vieron afectadas ^17^, y el uso de antibióticos fue excesivo pudiendo afectar en los mecanismos de resistencia.
